# Temperature of the Body in Health

**Published:** 1869-04

**Authors:** 


Temperature of the Body in Health.—Dr. Sydney Ringer gives an abstract of a paper lately laid before the Royal Society on this subject. He gives the results of the experiments made by himself and the late A. P. Stewart. The following are the conclusions. The average maximum temperature of the day in persons under 25 years of age is 99°·1 Fahr.; of those over 40, 98°·8 Fahr. There occurs a diurnal variation of the temperature, the highest point of which is maintained between the hours of 9 A. M. and 6 P. M. At about the last-named hour the temperature slowly and continuously falls, till, between 11 P. M. and 1 A. M., the maximum depression is reached. At about 3 A. M. it again rises, and reaches very nearly its highest point by 9 A. M. The diurnal variation in persons under 25 amounts, on an average, to 2°·2 Fahr.; but in persons between 40 and 50 it is very small, the average being not greater than 0°·87 Fahr.; nay, on some days no variation whatever happens. In these elderly people the temperature still further differs from that of young persons; for in the former the diurnal fall occurs at any hour, and not, as is the case with young persons, during the hours of night. Concerning the influence of food on the temperature of the body, the authors have concluded that none of the diurnal variations are in any way caused by the food we eat. The experiments to prove this conclusion are very numerous. Some were made with the breakfast, others with the dinner and tea; but all point to the conclusion just stated. This important question is very fully discussed in the section devoted to it. By cold baths both the surface of the body and the deep parts were lowered in temperature. The temperature of the surface was in some instances reduced to 88° Fahr.; but the heat so soon returned to all parts as to show that the cold bath is of very little use as a refrigerator of the body. The cold bath produced no alteration in the time or amount of the diurnal variation. This began at the same hour, and reached the same amount as on those days when no bath was taken. By hot-water or vapor baths the heat of the body could be raised very considerably. Thus, on some occasions, when using the general hot bath, the temperature under the tongue was noted to be between 103° and 104° Fahr.—a fever temperature. The body being heated considerably above the point at which combustion could maintain it, it was then shown with what rapidity heat may be lost, simply by radiation and evaporation. The experiments tend to prove that hot baths in no way affect the diurnal variation of the temperature.

